# Variations in policies for accessing elective musculoskeletal procedures in the English National Health Service: A documentary analysis

**DOI:** 10.1177/13558196221091518

**Published:** 2022-05-15

**Authors:** Leila Rooshenas, Sharea Ijaz, Alison Richards, Alba Realpe, Jelena Savovic, Tim Jones, William Hollingworth, Jenny L Donovan

**Affiliations:** 1 Population Health Sciences, Bristol Medical School, 1980 University of Bristol, UK; 2 The National Institute for Health Research Applied Research Collaboration West (NIHR ARC West), 1984 University Hospitals Bristol and Weston NHS Foundation Trust, UK

**Keywords:** Unwarranted variation, health policies, commissioning, surgical procedures

## Abstract

**Objective:**

The overall aim of this study was to investigate how commissioning policies for accessing clinical procedures compare in the context of the English National Health Service. Our primary objective was to compare policy wording and categorise any variations identified. Our secondary objective was to explore how any points of variation relate to national guidance.

**Methods:**

This study entailed documentary analysis of commissioning policies that stipulated criteria for accessing eight elective musculoskeletal procedures. For each procedure, we retrieved policies held by regions with higher and lower rates of clinical activity relative to the national average. Policies were subjected to content and thematic analysis, using constant comparison techniques*.* Matrices and descriptive reports were used to compare themes across policies for each procedure and derive categories of variation that arose across two or more procedures. National guidance relating to each procedure were identified and scrutinised, to explore whether these provided context for explaining the policy variations.

**Results:**

Thirty-five policy documents held by 14 geographic regions were included in the analysis. Policies either focused on a single procedure/treatment or covered several procedures/treatments in an all-encompassing document. All policies stipulated criteria that needed to be fulfilled prior to accessing treatment, but there were inconsistences in the evidence cited. Policies varied in recurring ways, with respect to specification of non-surgical treatments and management, requirements around time spent using non-surgical approaches, diagnostic requirements, requirements around symptom severity and disease progression, and use of language, in the form of terms and phrases (‘threshold modifiers’) which could open up or restrict access to care. National guidance was identified for seven of the procedures, but this guidance did not specify criteria for accessing the procedures in question, making direct comparisons with regional policies difficult.

**Conclusions:**

This, to our knowledge, is the first study to identify recurring ways in which policies for accessing treatment can vary within a single-payer system with universal coverage. The findings raise questions around whether formulation of commissioning policies should receive more central support to promote greater consistency – especially where evidence is uncertain, variable or lacking.

## Introduction

Health services worldwide are under pressure to provide high quality, equitable care, in the context of limited budgets, evolving evidence and costly innovations. Monitoring geographic variations in health care provision is a starting point for critically assessing current practice.^1^ These variations may be ‘warranted’ or ‘unwarranted’. Warranted variation refers to expected patters of clinical activity based on population need, patient preferences or innovation (where new treatments/procedure take time to diffuse). For example, variation in rates of emergency admission of febrile infants may be considered warranted if explained by differences in clinical presentation.^2^ Unwarranted variation, by contrast, cannot be explained in terms of patient preferences or needs, and can reflect differences in how health care systems operate and perform.^3,4^ Unwarranted variation can signify underuse or overuse, both of which have implications for equitable high-quality care and judicious use of resources.^1,3^ So if variation in emergency admissions for febrile children varied (once clinical presentation, age and population size had been accounted for), there may be grounds to suspect differences in the quality and efficiency of services or clinicians’ practices.

The persistence of unwarranted variation has been empirically documented in many high-income countries’ health care systems,^5–7^ but research into understanding and addressing these patterns is still evolving.^3,7^ Health localities may perform differently due to a multitude of factors, from differences in concentration of skills and workforce, to differences in clinical cultures and preferences - described by Wennberg as ‘practice style factors’.^8(p7)^ Clinical beliefs are known to drive variation, but individual clinicians’ judgements are typically situated within a wider context of regulation and policy, often mediated by ‘purchasers’ responsible for resource allocation. Purchasers’ roles can vary from being ‘allocators of funds’, to strategic decision-makers who determine the scale and nature of services in an area or organisation.^9^ The literature focuses heavily on clinician behaviours as a source of variation, but there has been comparatively less attention on purchasers’ influences. The literature has shown that those tasked with health care purchasing can access an array of knowledge/evidence types that extend beyond academic research, and that local data/evaluations and expert views can take precedence over formal research-based evidence.^10,11^ There is untapped potential to address practice variations through better understanding purchasers’ actions and outputs.

The English National Health Service (NHS) is a tax-funded single-payer system, with a purchaser-provider split in the commissioning and delivery of health care.^9^ Two-thirds of the health budget is managed by around 100 regional statutory bodies named Clinical Commissioning Groups (CCGs).^12^ CCGs are responsible for purchasing health care services (e.g. hospital procedures) on behalf of their local populations, with some autonomy over regulating clinical activity. One way in which they can do this is through enforcement of threshold policies (referred to as ‘commissioning policies’ in this article). These policies stipulate criteria that need to be fulfilled for a patient to be referred/listed for treatment. Audits of commissioning policies for several procedures have shown that access criteria can vary, but these insights are limited to a few studies highlighting clinical-specific criteria, limiting opportunities for transferability.^13–17^

The aim of this study was to identify ways in which commissioning policies for accessing clinical procedures can vary, irrespective of clinical context. Rather than reporting the scale of differences, we sought to generate new insights into *how* policies vary. Our secondary objective was to examine policies in relation to national guidance (if available), with a view to better understanding any source of discrepancies between regions.

## Methods

### Design

This was a documentary analysis of regional commissioning policies and national guidance for a sample of eight elective musculoskeletal procedures.

### Context

The ‘regions’ referred to in this study are located across England and were demarcated by ‘Sustainability and Transformation Partnerships’ (STPs). STPs provide strategic oversight of how care is integrated and delivered across CCGs in a given region. Our starting point for the study was to examine how one local STP’s expenditure for a sample of surgical procedures compared with the national average. We refer to this region as the ‘index-region’. The research team came to work with the index-region through an NIHR-funded Collaboration for Leadership in Applied Health Research and Care project. The researchers conducted a benchmarking exercise, to identify areas of clinical activity that the index-region was performing more frequently than the national average. The present study was conceived to explore if policies for accessing the procedures differed to those held by other regions, as a means of identifying a potential contributor to variation. Sampling decisions around selection of clinical procedures and other geographic regions were made in relation to the index-region, as follows.

### Selection of clinical procedures

We identified a list of procedures to serve as focal points for cross-policy comparisons. This selection was informed by the index-region’s strategic priorities and the benchmarking exercise described above. Musculoskeletal services had been identified as a high priority area for the index-region, as procedure rates were historically higher than the national average. We thus focussed on the top 10 musculoskeletal activities for which the index-region was considered ‘higher spend’ relative to the national average, based on Hospital Episode Statistic data adjusted for population differences (age, sex, Index of Multiple Deprivation scores (for 2015) and ethnicity (% white British)). The data were accessed via a licence from NHS Digital (DARS-NIC-17875-X7K1V). The procedures selected were hip arthroscopy, hip replacement, knee arthroscopy, knee replacement, rotator cuff repair (shoulder procedure), subacromial decompression (shoulder procedure), surgery for Dupuytren’s contracture (a hand condition, where one or more fingers bend towards the palm) and surgery for trigger finger (a hand condition, characterised by difficulty bending fingers or the thumb).

Further details on how we identified these procedures are available in the online Supplementary Material S1 and S2.

### Identification of other regions’ policies for comparison

We adopted a systematic approach to sampling at least three commissioning policies for each procedure: the index-STP’s policy (if this existed), a policy from a ‘high spend’ region, and a policy from a ‘low spend’ region, relative to the national average. STPs were ranked from lowest to highest spend for each procedure. We searched for policies held by the index-region, the lowest spend, and the highest spend region, by consulting STPs’ (or their constituent CCGs’) websites. If no policies were retrieved, this was documented, and the above steps were repeated for the next highest/lowest spend region. Other regional policies incidentally identified were also included in the analysis. We ensured that the policies identified for any given procedure had all been retrieved (or were still ‘live’) on the same day. We first retrieved the documents and began our analysis in August 2018. We reviewed the latest policy criteria after our analyses were complete in March 2020, and found most policies’ criteria were unchanged, with the categories of variation reported below unchanged.

The National Institute for Health and Care Excellence (NICE) is an independent agency that provides national guidance and advice to improve health and social care in the United Kingdom.^18^ Two members of our study team – an Information Scientist (ARi) and Systematic Reviewer (SI) – searched the NICE and NICE Clinical Knowledge Summaries^18^ websites to identify national guidance relating to each procedure. This guidance was sought for contextual purposes, to better understand any cross-policy variations.

### Analysis

We identified 35 policy documents from 14 different regions (STPs) for analysis (Table 1). One region (Region 4) had policies from different sub-regions (demarcated by CCGs), which were included in the analysis and labelled as ‘Region 4a’ and ‘Region 4b’. We also identified 22 NICE documents outlining potentially relevant national guidance, covering all but one of the procedures (surgical treatment of trigger finger).

**Table 1.** Policies and national institute of health and care excellence guidance identified for each clinical procedure.

Clinical procedure	Number of policies identified	Potentially relevant NICE guidance consulted
Hip arthroscopy	4 policies 1. Region 1 (index-region) 2. Region 6 3. Region 4a (a CCG within Region 4) 4. Region 4b (combined policy for multiple CCGs within Region 4)	1. NICE IPG408: Arthroscopic femoroacetabular surgery for hip impingement syndrome (Sep 2011)
Hip replacement	5 policies 5. Region 1 (index-region) 6. Region 8 7. Region 9 8. Region 10 9. Region 3	2. NICE CG177: Osteoarthritis: Care and management (Feb 2014) 3. NICE IPG363: Minimally invasive total hip replacement (Oct 2010) 4. NICE TA304: Total hip replacement and resurfacing arthroplasty for end-stage arthritis of the hip (Feb 2014)
Knee arthroscopy	5 policies 10. Region 1 (index-region) 11. Region 3 12. Region 4a (a CCG within Region 4) 13. Region 5 14. Region 2	5. NICE CG177: Osteoarthritis: Care and management (Feb 2014) 6. NICE IPG493: Arthroscopic radiofrequency chondroplasty for discrete chondral defects of the knee (May 2014) 7. NICE IPG162: Mosaicplasty for knee cartilage defects (March 2006) 8. NICE IPG430: Partial replacement of the meniscus of the knee using a biodegradable scaffold (July 2012) 9. NICE IPG474: Arthroscopic trochleoplasty for patellar instability (Jan 2014) 10. NICE IPG560: Microstructural scaffold (patch) insertion without autologous cell implantation for repairing symptomatic chondral knee defects (June 2016) 11. NICE IPG230: Arthroscopic knee washout, with or without debridement, for the treatment of osteoarthritis (Aug 2007)12. NICE TA477: Autologous chondrocyte implantation for treating symptomatic articular cartilage defects of the knee (Oct 2017) 13. NICE TA508: Autologous chondrocyte implantation using chondrosphere for treating symptomatic articular cartilage defects of the knee (March 2018) 14. NICE MIB30: LARS for reconstructing damaged intra-articular cruciate knee ligaments (May 2015)
Knee replacement	3 policies 15. Region 1 (index-region) 16. Region 3 17. Region 7	15. NICE CG177: Osteoarthritis: Care and management (Feb 2014) 16. NICE IPG345: Mini-incision surgery for total knee replacement (May 2010)
Rotator cuff repair	3 policies 18. Region 1 (index-region) 19. Region 2 20. Region 14	17. NICE clinical knowledge summary: Shoulder pain (scenario: Rotator cuff disorders) (April 2017) 18. (NICE-accredited) British Elbow & Shoulder Society (BESS), British Orthopaedic Association (BOA), Royal College of Surgeons for England (RCSEng) commissioning guide: Subacromial shoulder pain (2014)
Subacromial decompression	4 policies 21. Region 1 (index-region) 22. Region 11 (combined policy for multiple CCGs within Region 11) 23. Region 12 24. Region 2	19. (NICE-accredited) British Elbow & Shoulder Society (BESS), British Orthopaedic Association (BOA), Royal College of Surgeons for England (RCSEng) commissioning guide: Subacromial shoulder pain (Nov 2014)
Surgery for Dupuytren’s contracture	6 policies 25. Region 1 (index-region) 26. Region 3 27. Region 4a (a CCG within Region 4) 28. Region 4b (combined policy for multiple CCGs within Region 4) 29. Region 2 30. Region 6	20. NICE clinical knowledge summary: Dupuytren’s disease (Nov 2015) 21. NICE TA459: Collagenase clostridium histolyticum for treating Dupuytren’s contracture (July 2017) 22. NICE IPG43: Needle fasciotomy for Dupuytren’s contracture (Feb 2004)
Surgery for trigger finger	5 policies 31. Region 1 (index-region) 32. Region 3 33. Region 4b (combined policy for multiple CCGs within Region 4) 34. Region 13 35. Region 2	No NICE guidance identified

NICE: National Institute for Health and Care Excellence; IPG: Interventional Procedure Guides; CCG: Clinical Commissioning Groups.

All policies had at least one review date scheduled over the course of the study. Some policies had changed by 2020, prompted by introduction of national policy criteria for select procedures.^19^ Full details of policy dates, iterations and changes to criteria are documented in online Supplementary Material S3 and S4).

The policies identified for each procedure were imported into NVivo (version 10) and analysed thematically, using constant comparative approaches.^20^ This involved coding the policy documents line-by-line and iteratively arranging the codes into thematic categories. Three researchers (LR, AR and SI) independently coded two policies for two procedures, and met to discuss their coding. Policy content had been interpreted similarly, although there were differences in levels of coding detail. The team agreed that sections of policy that described *criteria for treatment or referral* should be coded in depth, in line with the study aim. One researcher (LR) coded the remainder of the policies in line with the above. The coding framework evolved as new policies were analysed and compared with previously coded documents. This was regularly discussed with other members of the team in data interpretation meetings.

Extracts from coded policies were inputted into matrices (one matrix per procedure) to enable cross-comparisons of different commissioning policies. The matrix was populated with descriptive text and verbatim policy extracts relating to each theme. Descriptive summaries of similarities/differences across policy criteria were written for each theme. Data were then synthesised in an overarching matrix, listing the types of policy variation identified against the procedures (online Supplementary Material S3). This was populated with summaries of how policies compared, supported with verbatim extracts. This overarching matrix formed the basis of a final description report, which detailed the types of variation identified between policies, and how frequently these arose across the procedures.

To address the secondary objective, we conducted content analysis on the relevant NICE guidance documents identified for each procedure. This entailed searching these documents for text that provided clarity around the points of variation identified in the cross-policy analyses. Relevant text was pasted or summarised in the overarching matrix mentioned above (online Supplementary Material S3).

## Results

### Broad content, structure and remit of commissioning policies

All policies communicated criteria that patients needed to fulfil prior to accessing the procedures. A variety of terms were used to describe these policies: most were neutral descriptors (e.g. ‘commissioning policy’), but some indicated a rationale for restricting access to the procedures they discussed. The title for Region 12’s policies, for example, was suggestive of promoting judicious resource use (‘Effective use of resources policy’), whilst Region 13’s policies were framed as limiting procedures of ‘limited clinical value’ (not defined in this document). Where resources were discussed in policies from six regions, this was mentioned in the context of the commissioning body’s responsibility to ensure funding decisions about treatment provision were based on evidence considerations, cost-effectiveness, and/or maximising health benefits from the resources available (policies from Regions 4a, 4b, 5, 7, 8, 12).

Eight regions’ policies focused on a single procedure/treatment (those for Regions 1, 2, 3, 6, 9, 11, 12 and 14). The remainder covered several procedures/treatments in an all-encompassing document (Regions 4a/b, 5, 7, 8, 10 and 13). Some policies included information about the procedure, whilst others solely listed criteria for treatment/referral. Policies also varied in their engagement with scientific literature, ranging from detailed summaries of the evidence with citation lists, to no citations at all (six policies, from Regions 5, 6, 7, 9, 10 and 13). A detailed, side-by-side comparison of reference lists is shown in online Supplementary Material S5.

We compared citation lists from policies relating to each procedure and identified considerable variation, to the extent that no two reference lists were the same. This partly reflected differences in policy content (e.g. whether background information was included in a policy), but there were also inconsistencies in whether clinical guidance and comparative-effectiveness evidence (e.g. systematic reviews/randomised control trials) appeared across policies. For example, each of the four regions’ policies for subacromial decompression (Regions 1, 2, 11 and 12) were different in almost every way. There were no citations common to all four regions’ policies. The only similarities comprised references to a national, NICE-accredited commissioning guide (mentioned in Regions 1 and 12, but absent in Regions 2 and 12), and two randomised studies (mentioned in Regions 11 and 12, but absent in Regions 1 and 2). Citations were mostly unique to each policy, with each region citing at least one systematic review and primary study that was not mentioned in any other policy. Overall, comparison of citation lists indicated that policies had not drawn upon the same publications, despite all being the most up-to-date policies retrieved from STPs’/CCGs’ websites.

### Differences in threshold criteria for accessing surgical procedures

Variations in criteria for treatment and/or referral were identified across policies for all procedures examined. Five types of variation arose across policies for most procedures. These included differences in requirements around non-surgical treatment/management, time spent using non-surgical approaches, diagnostic requirements, symptom severity and disease progression, and differences in use of ‘threshold modifiers’. The categories of variation are discussed in the sub-sections below.

### Differences in requirements around non-surgical treatment/management

Variations in specification of non-surgical treatment/management were apparent for every procedure, apart from surgical treatment of Dupuytren’s contracture. Policies for some procedures were similar in that they mentioned non-surgical management, often referred to as ‘conservative’ care, in some capacity (e.g. hip arthroscopy, knee replacement, hip replacement and subacromial decompression). By contrast, policies for knee arthroscopy, surgical treatment of trigger finger and rotator cuff repair did not mention non-surgical management consistently.

The most striking example of variation was apparent across knee arthroscopy policies, where there were inconsistencies in whether non-surgical management was mentioned at all (mentioned in Regions 1, 2, 4a and 5, but not mentioned in Region 3). Differences were also apparent in whether the type of non-surgical management was specified. For example, Regions 1 and 2 gave specific examples of non-surgical management (Region 1: ‘lifestyle advice, optimum pharmacological treatments rest, self or physiotherapy guided mobilisation and strengthening exercises’; Region 2: ‘can include advice, physio and support from the musculoskeletal services and pain management with non-steroidal anti-inflammatory drug (NSAID) painkillers’.). Region 4a and 5 did not provide this level of detail.

Where non-surgical management was defined, this too could vary. Policies for surgical treatment of trigger finger were inconsistent in whether they branded injections and splinting as ‘conservative care’. Policies for hip arthroscopy also defined ‘conservative’ treatment variably. Three regions defined this as non-specialist activity modification, physiotherapy and pharmacological intervention (Region 4a, Region 4b and Region 6), but one region’s policy (Region 1) only mentioned activity modification, restriction of exercise and avoidance of symptomatic motion. Here is an extract from Region 4a’s hip arthroscopy policy, detailing the conditions under which hip arthroscopy would be provided:

The symptoms have not responded to all available conservative treatment options including activity modification, drug therapy (NSAIDs) and specialist physiotherapy.

By contrast, here is the corresponding passage in Region 1’s policy:

The patient has fully engaged with conservative therapy for at least 3 months including activity modifications, restriction of exercise and avoidance of symptomatic motion.

Variations in descriptions of non-surgical management were also identified, in terms of which (if any) conservative approaches were mandatory. Taking subacromial decompression as an example, all regions’ policies mentioned ‘rest’, ‘activity modification’, ‘pharmaceutical therapy’, ‘physiotherapy’ and ‘steroid injections’, but there were differences in which of these were branded essential. Region 1 stipulated steroid injections as essential, preceded by any other form of conservative care. Region 12 also presented injections as essential, albeit following treatment with all other forms of conservative care. Region 2 singled out physiotherapy as essential, but did not require patients to receive a steroid injection. Similarly, Region 11 included a clause indicating that injections only needed to be administered if deemed ‘appropriate’ (subjective clauses are discussed below). Similar variations around conservative care requirements were identified across policies for rotator cuff repair and knee arthroscopy (online Supplementary Material S3).

### Differences in required time spent using non-surgical approaches

Policies for seven procedures differed in terms of the length of time patients needed to have spent trying non-surgical management. For example, knee arthroscopy criteria ranged from ‘at least 3 months’ (Region 1) to 12 months (Region 2) of non-surgical management, and subacromial decompression and rotator cuff repair policies ranged from 6 weeks (Region 1) to 9 months (Region 2). Variations in time requirements were also observed for hip and knee replacement, surgical treatment of trigger finger, and hip arthroscopy policies.

Discrepancies also arose around whether treatment duration was specified or not. In the three policies compared for knee replacement, one region specified 6 months of conservative treatment (Region 1), one specified 3 months (Region 7), and one did not state a duration (Region 3). These discrepancies were also observed in policies for hip and knee arthroscopy, trigger finger and hip replacement. For instance, here are extracts from different regions’ hip replacement policies:

Region 1: Referral to secondary care and subsequent treatment may be provided where… [the] patient has:

-Fully engaged with conservative measures for a period of at least six months…as detailed within this policy, and this has failed to improve the symptoms of the patient

Region 10: Treatment will be supported when:…[the] patient has experienced persistent severe relevant pain despite adequate or maximally tolerated management in the primary and/or community setting

Region 10: Referral for specialist assessment should only be considered if the patient has: Moderate to severe pain not adequately relieved by an extended course of non-surgical treatment (such as adequate doses of analgesia, weight control and physical therapies) and [other factors]

In a similar vein, policies for surgical treatment of trigger finger differed in their specification of how many rounds of steroid injections patients needed to undergo: ‘at least one’ injection was required in three regions (Regions 1, 2, and 12), ‘at least 2’ in Region 3, and ‘at least two, followed by ultrasound scan with or without a further corticosteroid injection’ in Region 4b (online Supplementary Material S3).

### Differences in diagnostic requirements

Policies for six procedures differed in terms of diagnostic requirements, including investigations (e.g. imaging tests) and confirmation of clinical diagnoses.

Policies for five procedures varied in their requirements for diagnostic investigations (knee replacement, hip replacement, trigger finger, subacromial decompression and rotator cuff repair). Some knee and hip replacement policies specified that patients needed to have ‘radiographic evidence’ of disease, for instance. Taking surgical treatment of trigger finger as an example, Region 4b’s policy required patients to undergo an ultrasound scan (Supplementary Material S3), whilst other regions made no reference to such tests. Policies for subacromial decompression and rotator cuff repair also varied in their specification of diagnostic imaging. Of the four policies for subacromial decompression (Regions 1, 11, 2 and 12), only one (Region 12) recommended an x-ray to confirm impingement. Similarly, of three policies compared for rotator cuff repair (Regions 1, 2 and 14), only one region’s policy recommended an MRI or ultrasound scan to confirm the muscle tear (Region 2). By contrast, diagnostic imagining requirements were consistent for knee arthroscopy: all policies from Regions 1, 2, 3, 4a and 5 permitted either clinical examination or diagnostic imagining to confirm internal joint derangement. Hip arthroscopy policies were also consistent, in that all regions required radiographic evidence of femoroacetabular impingement (Regions 1, 4a, 4b and 6).

Variations arising across knee arthroscopy policies were distinct, as these related to whether the procedure itself could be used as a diagnostic investigation. Policy statements ranged from stating knee arthroscopy would not be funded for diagnostic purposes (Region 1 and Region 4a), through to specification of some scenarios where this would be permitted (Region 3 and Region 5):

Region 1: Knee arthroscopy is not routinely commissioned…for diagnostic purposes only.

Region 4a: Knee arthroscopy should NOT be carried out for…investigation of knee pain (MRI is a less invasive alternative)

Region 3: Use of knee arthroscopy as a diagnostic tool will only be funded in the following situations:

- Patients with medial knee pain where the Plica syndrome is suspected.

- When Chondromalacia patellae is suspected

Region 5: [Knee arthroscopy will only be funded when] there is continuing diagnostic uncertainty following MRI, such that a Consultant Knee Surgeon recommends diagnostic arthroscopy.

Policies for three procedures consistently mentioned diagnosis of particular conditions in their criteria (knee arthroscopy policies mentioned ‘joint derangement’, hip arthroscopy policies mentioned ‘femoroacetabular impingement’, subacromial decompression policies mentioned ‘impingement’), but other policies exhibited variation on this front. These variations had potential to tighten or expand opportunities for accessing surgery. For example, Region 1’s policy for knee replacement specified that patients needed to have been diagnosed with end-stage osteoarthritis, but no other policy stipulated this. Region 3’s policy referred to osteoarthritis in the ‘background’ section of its policy, explaining that this was a common (but not the only) indication for knee replacement: ‘Total knee replacement can be performed for a number of conditions, but it is most often for osteoarthritis of the knee.’

Region-1’s policy for surgical treatment of trigger finger stipulated that patients with inflammatory arthritis could bypass other pre-requisites for surgery, including conservative treatment requirements:

The CCG will agree to fund surgical intervention for trigger finger where the: (1). Patient has been diagnosed with inflammatory arthritis; AND (2). There is a joint agreement by the patient’s Rheumatoid Arthritis Consultant and Hand Surgeon that their trigger finger is unlikely to be corrected by conservative treatment.

This clause appeared to lower the threshold for surgery, but did not appear in any other policies.

Though only apparent in rotator cuff surgery policies, discrepancies in diagnostic criteria could also relate to specificity. Policies for this procedure were inconsistent in their reference to ‘full’ or ‘partial’ tears; some made this distinction (Regions 2 and 14), and one did not (Region 1).

### Differences in specification of symptom severity and disease progression

Differences in specification of symptom severity/disease progression arose across policies for all procedures examined. For some procedures, policies across all regions consistently referred to severity descriptors, such as those for hip arthroscopy, which required patients to have ‘severe symptoms of femoroacetabular impingement’. Not all procedures shared this uniformity. Discrepancies in symptom severity were observed in surgical treatment of trigger finger, hip replacement, and knee replacement policies. Region 2 and Region 4b’s policies for trigger finger, for example, referred to specific levels of severity, although these terms were only defined in Region 2:

Region 2: The patient has moderate symptoms as defined below, which have not improved following conservative treatment, eg encouragement to regularly move the finger, rest from aggravating activities, splinting, NSAIDs, and at least one corticosteroid injection (unless contraindicated).

OR

The patient has severe symptoms as defined below that cannot be corrected with any other method.

Region 4b: Moderate to severe symptoms ongoing for at least 2 months not responding to conservative treatment.

Other regions’ policies did not refer to these grades of severity at all, instead just referring to a ‘fixed flexion deformity’ (Regions 1, 3 and 13).

Policies for hip and knee replacement surgery demonstrated variation in definitions of severity, despite consistency in other aspects. Policies were similar, in that they consistently referred to degrees of pain and functional impairment as ‘mild’, ‘moderate’, ‘intense’ and ‘severe’. Not all policies referred to the full range of terms, and definitions of these terms (if provided) were inconsistent. Taking hip replacement policies as an example, Region 1 mentioned four classification systems for pain (‘slight’, ‘moderate’, ‘intense’ and ‘severe’), whilst Region 3 stated three classifications (‘mild’, ‘moderate’ and ‘severe’). Although terms were common to both policies, their definitions differed (Supplementary Material S3). There was no alignment between any of the definitions of pain severity between these two policies.

Unlike other procedures, policies for Dupuytren’s contracture consistently mentioned objective markers of severity, in terms of the degree (angle) of flexion deformity at different joints. Variations in these policies arose in relation to the degree and location of the flexion (see online Supplementary Material S6 for extracts, discussed more fully in next section).

### Threshold modifiers

Policies often included clauses or statements that could tighten or relax thresholds for surgery, referred to here as ‘threshold modifiers’. Even if policies had adopted the same criteria, threshold modifiers carried potential to expand or restrict routes to accessing surgery. Threshold modifiers introduced variation across policies for every procedure examined. Two types of threshold modifiers arose in the analysis: ‘AND/OR terms’ and ‘get-out-clauses’.

First, thresholds could be modified based on how ‘AND’/’OR’ terms were used between listed policy criteria, as illustrated through policies for surgical treatment of Dupuytren’s contracture (Supplementary Material S6). All policies mentioned that patients needed to have a flexion deformity at one or two finger joints. There was clear variation in the degree of deformity specified in policies (variation in severity), but policies also carried different meanings arising from how ‘AND/OR’ were used. Region 1’s policy stated that patients needed to have a flexion deformity of ‘≥30°’ at either joint, in addition fulfilling at least one other criteria relating to functioning, progression of disease, or impact on lifestyle (‘AND’ operator). By contrast, Region 3’s policy suggested patients could have a less severe deformity, as long as they fulfilled one other criterion relating to progression of disease or impact on lifestyle. Region 6’s use of ‘OR’ between all criteria appeared to expand opportunities for surgery, even if patients did not have a flexion deformity. Region 2’s policy appeared to be the most stringent, in that having a deformity was an essential criterion for both joints, with no other routes for accessing surgery.

Threshold modifiers could also take the form of ‘get-out clauses’: statements that allowed patients to bypass criteria. These clauses appeared inconsistently across policies for all procedures examined. Clauses sometimes referred to clinical diagnoses that allowed patients to bypass other criteria (as described earlier), but they were often subjective, inviting clinician judgement. These statements could be subtle. For example, Region 11’s policy for subacromial decompression included the words ‘where clinically appropriate’ next to a criterion about steroid injection, in contrast to policies from other regions that stated injection was a requirement (Regions 1, 2 and 12). A policy for knee arthroscopy (Region 5) similarly deviated from others (Regions 1, 2, 3 and 4a), by stating that conservative treatment could be bypassed ‘where it is clear that conservative treatment will not be effective’.

Some get-out clauses were standalone statements, appearing separate to a policy’s other criteria. Region 5’s policy for knee arthroscopy, for instance, indicated that the criteria listed could be bypassed if: ‘intractable knee pain [is] considered likely to benefit from arthroscopic treatment according to assessment by a Consultant Knee Surgeon’. No similar statements appeared in other regions’ policies. Similar variations were apparent in hip arthroscopy policies. Region-1 and Region 6 specified that patients could access treatment if they were judged to require urgent treatment, as this extract from Region 6 shows:

Region 6 policy for hip arthroscopy: [Hip arthroscopy will be funded if patients have] Compromised function, which requires urgent treatment within a 6–8 months time frame, or where failure to treat early is likely to significantly compromise surgical options at a future date.

No such statements appeared in other regions’ policies

Policies for knee replacement and hip replacement also varied in the use of clauses around clinicians’ judgement about the expediency of surgery. Only one hip replacement policy, that of Region 1, included a statement that permitted surgery to proceed, if delaying was thought to lead to a technically more challenging future operation: ‘The patient is at risk of destruction of their joint of such severity that delaying surgical correction would increase the technical difficulties of the procedure’.

### Comparisons between regional policies and national institute for health and care excellence guidance

NICE clinical guidance tended not to include information relevant to the categories of variation identified above, in that the guidance generally did not specify detailed thresholds for treatment; rather, the guidance presented information about appropriate options that *could* be implemented, in line with clinician judgement. The NICE clinical guidance for management of osteoarthritis, for example, stipulated that the recommendations should be considered in tandem with individual patient circumstance and local and national priorities:

NICE Clinical Guideline 177^21^: It is not mandatory to apply the recommendations, and the guideline does not override the responsibility to make decisions appropriate to the circumstances of the individual, in consultation with them and their families and carers or guardian. Local commissioners and providers of health care have a responsibility to enable the guideline to be applied when individual professionals and people using services wish to use it. They should do so in the context of local and national priorities for funding and developing services

NICE Interventional Procedure Guides (IPGs) also had a standing statement establishing their role as providing safety and efficacy recommendations, rather than cost-effectiveness and uptake (e.g. IPG363, IPG493, IPG162, IPG430, IPG474, IPG560, IPG230, IPG408, IPG43 and IPG345 in Table 1). Similarly, NICE Technology Appraisals (TAs) mandated commissioning organisations to make certain procedures available, but did not specify criteria for accessing these (e.g. TA304, TA477, TA508 and TA459 in Table 1).

The commissioning policies did not contradict NICE guidance, but were more specific and detailed in their specification of thresholds for treatment. For example, hip and knee procedure policies referred to some or all of the conservative treatments mentioned in the NICE clinical guidance on management of osteoarthritis, but some went further by articulating which of these were essential, and/or set minimum durations for treatment.

A NICE-accredited commissioning guide for subacromial shoulder pain by the British Elbow & Shoulder Society, British Orthopaedic Association and Royal College of Surgeons for England^22^ (Table 1) included information that was more comparable with the commissioning policies for subacromial decompression and rotator cuff repair. The guide specified types of conservative care that needed to be attempted, recommended durations of conservative care, types of diagnostic investigations required and courses of action dependent on symptoms. The commissioning guide appeared in two policies for subacromial decompression (from Regions 1 and 12) and two policies for rotator cuff repair (from Regions 1 and 14), but none of the policies wholly aligned with the guidance, deviating according to one or more of the categories described above. Similar to NICE guidance, however, this commissioning guide emphasised judgement in implementing its recommendations ‘can be modified according to the needs of the local health economy’.^22^

## Discussion

This, to our knowledge, is the first study to identify common ways in which commissioning policies stipulating criteria for accessing treatments can vary. Policies varied in recurring ways, including specification of non-surgical treatment/management, requirements around time spent using non-surgical approaches, diagnostic requirements, and requirements around symptom severity and disease progression. The use of particular terms and phrases – ‘threshold modifiers’ – were found to alter policy meaning by expanding or restricting opportunities for referral/treatment. Policies differed in their references to literature and clinical guidance, indicating lack of consistency over the sources of evidence they were based upon. Comparison with national clinical guidance did not illuminate which (if any) criteria in policies were most appropriate, in that they generally did not provide the level of detail and specificity articulated in the commissioning policy criteria.

Research examining devolved health care purchasers’ (commissioners’) practices can provide insight into reasons underpinning the policy variations identified. Previous studies have identified challenges in developing policies, including limited time and local skills/expertise to systematically identify and appraise evidence.^11,23,24^ Others have suggested that the evidence-culture within commissioning organisations differs to that of medicine, in that the source of evidence (e.g. local relevance) and mode of communication (e.g. practical guidance) lend more credence than the evidence hierarchy.^11^ The interpretation of evidence can also be a dynamic process, as information can be ‘juggled’ and ‘steered’ through meetings and chance encounters.^11^ This decision-making culture provides context that may help to explain the policy variations identified in this research.

An assumption held at the outset of this study was that criteria for accessing procedures *should* be similar across commissioning organisations, but findings from this research have challenged this. National guidance or research evidence may not exist at the level of detail/specificity articulated in the commissioning policy criteria. This is likely to be the case in this study, given the evolving (or absent) evidence-base for many of the musculoskeletal procedures examined.^25^

The assumption that commissioning policy criteria should be identical also overlooks the possibility that policy wording may be shaped by local resource considerations. There were references to financial considerations in the background sections of some of the policies examined, but it was unclear if these statements reflected overarching commissioning principles, or factors that *specifically* guided selection/formulation of the policies. Though limited to one study, previous research has revealed commissioners’ propensity to emphasise how policy criteria are driven by evidence, though clinical professionals suspect cost-saving motives.^26,27^ It is unlikely that *clinical criteria* are manipulated to manage activity/expenditure, but the principles and processes for formulating policies warrants further investigation, with consideration to transparency and how others (e.g. clinicians and patients/service users) interpret the policies.

Variation in commissioning policy criteria implies that the ease/speed by which patients can access procedures will depend on where they live. Although there are questions around whether (and how) policies affect provision of care, the baseline variation in policy criteria challenges notions of equitable care. There are questions around whether policies of this nature should continue to be formulated by devolved bodies.^17^ In England, the ‘evidence-based interventions' programme has sparked new momentum in developing centralised policy criteria for accessing care,^20^ and commissioning groups are now required to ‘pay due regard’ to these policies.^20(p141)^ A mixed-methods evaluation of this programme has just launched, to understand implications of the programme for commissioners, clinical professionals, and patients. This will include investigation of how the new centralised processes compare with practice prior to the evidence-based interventions programme, addressing uncertainties around the process and principles underpinning commissioning policy formulation and function.^28^

Even if there was uniformity in commissioning policies, practice variations will likely endure, due to the myriad of factors that can affect service provision. Differences in funding mechanisms/incentives, alternative forms of care, and diffusion of innovation can drive variation. There are also questions around how commissioning policies shape clinicians’ practices. The tiers of individual, organisational, and environmental factors that shape clinicians’ engagement with guidance are well understood,^29,30^ but commissioning policies may differ, by virtue of their composition by non-clinical bodies, and potential financial consequences of non-compliance. Previous research indicates that while policies cannot be ignored, clinicians can manoeuvre around them, though this work was only conducted in two regions of the NHS in relation to one procedure.^27^ The ongoing evaluation of the evidence-based interventions programme, due to report in 2023, will examine a range of commissioning policies and their impacts on front-line practice.^28^

## Limitations

The inductive nature of our analysis was a key strength, as it reduced the risk of the findings being constrained by preconceptions. The cross-procedural analysis was another strength, as it yielded new knowledge beyond clinical-specific contexts. However, our study does have two main limitations. First, there was our lack of communication with those involved in policy compilation. Although information scientists were employed to locate the most up-to-date publicly available policies, there is a possibility that the documents retrieved were artefacts, or the research team had simply not looked in the right place.

Second, our findings solely focused on the NHS context and musculoskeletal procedures. This limits the transferability of our results. Focussing on regional policies in one health care system was necessary for this study, but there is a need to examine the transferability of these findings to other health care systems. There is also a need to test whether the categories of variation are transferable to other clinical specialities and non-therapeutic interventions, such as diagnostic tests. Analysis of purchasers’ or non-clinical stakeholders’ policies for accessing care should be one component of future research around practice variations, but needs to be coupled with methods to understand the role of policies in influencing clinical decisions.

## Conclusions

This study has identified recurring ways in which commissioning policies around access to treatments can vary, irrespective of clinical context. Comparison with national clinical guidance did not illuminate which (if any) criteria in policies were most appropriate, likely reflecting limited evidence to guide these decisions. The findings raise questions around whether compilation of clinical criteria for accessing treatments should continue to be formulated at a devolved (regional) level. Central bodies may be better placed to lead on this, with input from clinical, commissioning and patient/public stakeholders.

Our findings have practical implications, in that commissioners or similar agents involved in regional policy compilation can consider each of the categories of variation, and whether they are applicable to their policies and those held by other regions/institutions. This, at the very least, can prompt justifications for differences.

Our findings also have wider implications, relating to who should be responsible for compiling clinical criteria stipulating access to treatments. In light of our findings, there is a strong argument for clinical criteria for accessing treatments to be centrally formulated (e.g. by NHS England and/or clinical speciality groups), with input from other clinicians, commissioners, and patient/service users. Even where evidence is lacking, this will help to maintain consistency in baseline criteria for accessing care.

## Supplemental Material

Supplemental Material - Variations in policies for accessing elective musculoskeletal procedures in the English National Health Service: A documentary analysis Click here for additional data file. Supplementary Material for Variations in policies for accessing elective musculoskeletal procedures in the English National Health Service: A documentary analysis by Leila Rooshenas, Sharea Ijaz, Alison Richards, Alba Realpe, Jelena Savovic, Tim Jones, William Hollingworth and Jenny Donovan in Journal of Health Services Research & Policy.

Supplemental Material - Variations in policies for accessing elective musculoskeletal procedures in the English National Health Service: A documentary analysis Click here for additional data file. Supplementary Material for Variations in policies for accessing elective musculoskeletal procedures in the English National Health Service: A documentary analysis by Leila Rooshenas, Sharea Ijaz, Alison Richards, Alba Realpe, Jelena Savovic, Tim Jones, William Hollingworth and Jenny Donovan in Journal of Health Services Research & Policy.

Supplemental Material - Variations in policies for accessing elective musculoskeletal procedures in the English National Health Service: A documentary analysis Click here for additional data file. Supplementary Material for Variations in policies for accessing elective musculoskeletal procedures in the English National Health Service: A documentary analysis by Leila Rooshenas, Sharea Ijaz, Alison Richards, Alba Realpe, Jelena Savovic, Tim Jones, William Hollingworth and Jenny Donovan in Journal of Health Services Research & Policy.

Supplemental Material - Variations in policies for accessing elective musculoskeletal procedures in the English National Health Service: A documentary analysis Click here for additional data file. Supplementary Material for Variations in policies for accessing elective musculoskeletal procedures in the English National Health Service: A documentary analysis by Leila Rooshenas, Sharea Ijaz, Alison Richards, Alba Realpe, Jelena Savovic, Tim Jones, William Hollingworth and Jenny Donovan in Journal of Health Services Research & Policy.

Supplemental Material - Variations in policies for accessing elective musculoskeletal procedures in the English National Health Service: A documentary analysis Click here for additional data file. Supplementary Material for Variations in policies for accessing elective musculoskeletal procedures in the English National Health Service: A documentary analysis by Leila Rooshenas, Sharea Ijaz, Alison Richards, Alba Realpe, Jelena Savovic, Tim Jones, William Hollingworth and Jenny Donovan in Journal of Health Services Research & Policy.

Supplemental Material - Variations in policies for accessing elective musculoskeletal procedures in the English National Health Service: A documentary analysis Click here for additional data file. Supplementary Material for Variations in policies for accessing elective musculoskeletal procedures in the English National Health Service: A documentary analysis by Leila Rooshenas, Sharea Ijaz, Alison Richards, Alba Realpe, Jelena Savovic, Tim Jones, William Hollingworth and Jenny Donovan in Journal of Health Services Research & Policy.
