# Supplementary material for: Variations in policies for accessing elective musculoskeletal procedures in the English National Health Service: A documentary analysis
Source: J Health Serv Res Policy. 2022 May 15;27(3):190–202. doi: 10.1177/13558196221091518 (PMC9277328; doi:10.1177/13558196221091518)
Supplement: Supplemental Material - Variations in policies for accessing elective musculoskeletal procedures in the English National Health Service: A documentary analysis [file sj-pdf-1-hsr-10.1177_13558196221091518.pdf]

Journal of Health Services Research & Policy
Variations in policies for accessing elective musculoskeletal procedures in the English National Health Service: A documentary analysis
Leila Rooshenas L, Ijaz S, Richards A, Realpe A, Savovic J, Jones T, Hollingworth W and Donovan J

SUPPLEMENT 1

S1: Procedure identification

Hospital Episode Statistic data is recorded as OPCS-4-coded clinical activities, rather than the procedure names presented in the main body paper. Each STP's activity rates for each OPCS-4 coded 'invasive therapeutic' activity were adjusted for population differences, based on age, sex, Index of Multiple Deprivation scores (for 2015), and ethnicity (% white British).[1] Financial spend associated with each OPCS-4 code was calculated using national tariffs, and STPs were ranked from lowest to highest expenditure for each code. The findings were incorporated into a database, which allowed generation of charts to display each STP's expenditure relative to the national average for each coded activity.

Musculoskeletal services had been identified as a high priority area for the index-STP. We thus focused on the top 10 musculoskeletal OPCS-4 codes for which the index STP was considered 'higher spend' relative to the national average **(S2, column 1).** Two Information Scientists (AR and SI) inputted each of the codes into a search engine to identify associated clinical procedures, and consulted with clinical experts where further clarification was needed. Eight of the 10 codes clearly linked to surgical procedures which became the 'case-study procedures' for the regional policy comparisons **(S2, column 2)**. These were: subacromial decompression (shoulder procedure); surgical repair of rotator cuff tears (shoulder procedure); knee arthroscopy; hip arthroscopy; knee replacement; hip replacement; surgical treatment for Dupuytren's contracture, and surgical treatment for trigger finger (both hand procedures). Two of the clinical codes - "other excision of bone" and "operations on bursa" - were too broad to link to a specific procedure, but appeared to be associated with some of the case-study procedures already identified (e.g. subacromial decompression, hip/knee arthroscopy).

References:

1. Jones T, Carr AJ, Beard D, et al. Longitudinal study of use and cost of subacromial decompression surgery: the need for effective evaluation of surgical procedures to prevent overtreatment and wasted resources. *BMJ open* 2019; 9: e030229-e030229. DOI: 10.1136/bmjopen-2019-030229.
