# Supplementary material for: Variations in policies for accessing elective musculoskeletal procedures in the English National Health Service: A documentary analysis
Source: J Health Serv Res Policy. 2022 May 15;27(3):190–202. doi: 10.1177/13558196221091518 (PMC9277328; doi:10.1177/13558196221091518)
Supplement: Supplemental Material - Variations in policies for accessing elective musculoskeletal procedures in the English National Health Service: A documentary analysis [file sj-pdf-2-hsr-10.1177_13558196221091518.pdf]

Journal of Health Services Research & Policy
Variations in policies for accessing elective musculoskeletal procedures in the English National Health Service: A documentary analysis
Leila Rooshenas L, Ijaz S, Richards A, Realpe A, Savovic J, Jones T, Hollingworth W and Donovan J

SUPPLEMENT 2

S2. History of searches undertaken for each procedure until a relevant policy was identified.

This table show a record of the searches undertaken to identify policies for each case-study procedure, starting with the index-region, the highest spend region, and the lowest spend region.

<table>
<tr><th>OPCS-4 code and procedure name searched</th><th>Types of procedures/conditions</th><th>Was there a policy held by the index-region?</th><th>Low spend regions searched</th><th>High spend regions searched</th></tr>
<tr><td rowspan="3">Excision of bone (Subacromial decompression)</td><td rowspan="3">Subacromial decompression</td><td rowspan="3">Yes</td><td>No policies identified for first 5 lowest spend regions</td><td rowspan="3">Policy identified for highest spend region</td></tr>
<tr><td>Policy identified for 6th lowest spend region</td></tr>
<tr><td>Policy opportunistically found for 8th lowest spend region</td></tr>
<tr><td>Hybrid prosthetic replacement of hip joint using cement (Primary hybrid prosthetic replacement of hip joint using cemented femoral component)</td><td>Hip replacement 1</td><td>Yes</td><td>Policy identified for lowest spend region</td><td>Policy identified for highest spend region</td></tr>
<tr><td>Therapeutic endoscopic operations on other</td><td>Knee arthroscopy 1</td><td>Yes</td><td>Policy identified for lowest spend region</td><td>Policy identified for highest spend region</td></tr>
</table>

<table>
<tr><td rowspan="3">articular cartilage (Endoscopic shaving of articular cartilage) (Endoscopic articular thermal chondroplasty)</td><td></td><td></td><td>Policy opportunistically identified for 5th lowest spend region</td><td></td></tr>
<tr><td rowspan="2">Hip arthroscopy</td><td rowspan="2">Yes</td><td>No policies identified for first three lowest spend regions</td><td rowspan="2">Policy identified for highest spend region</td></tr>
<tr><td>Policy identified for 4th lowest spend region</td></tr>
<tr><td rowspan="3">Repair of muscle (Plastic repair of rotator cuff of shoulder NEC*)</td><td rowspan="3">Rotator Cuff Repair</td><td rowspan="3">Yes</td><td>No policies identified for first three lowest spend regions</td><td rowspan="2">No policies identified for top two highest spend regions</td></tr>
<tr><td>Policies found for 4th lowest spend region (some CCGs)</td></tr>
<tr><td>Policy opportunistically found for 7th lowest spend region</td><td>Policy identified for 3rd highest spend region</td></tr>
<tr><td rowspan="2">Excision of other fascia (Palmar fasciectomy) (Digital fasciectomy)</td><td>Surgery for Dupuytren's contracture</td><td>Yes</td><td>Policy identified for lowest spend region</td><td>Policy identified for highest spend region</td></tr>
<tr><td>Surgery for Trigger finger</td><td>Yes</td><td>Policy identified for lowest spend region</td><td>Policy identified for highest spend region</td></tr>
</table>

Total prosthetic replacement of knee joint using cement (Primary total prosthetic replacement of knee joint using cement)	Knee replacement	**Yes**	**Policy identified for lowest spend region**	**Policy identified for highest spend region**
Total prosthetic replacement of hip joint not using cement (Primary total prosthetic replacement of hip joint not using cement)	Hip replacement 2	**Yes**	**Policy identified for lowest spend region**	**Policy identified for highest spend region**
Other reconstruction of ligament (Reconstruction of intra-articular ligament)	Knee arthroscopy 2	**Yes**	**Policy identified for lowest spend region**	**Policy identified for highest spend region**
