# Supplementary material for: Variations in policies for accessing elective musculoskeletal procedures in the English National Health Service: A documentary analysis
Source: J Health Serv Res Policy. 2022 May 15;27(3):190–202. doi: 10.1177/13558196221091518 (PMC9277328; doi:10.1177/13558196221091518)
Supplement: Supplemental Material - Variations in policies for accessing elective musculoskeletal procedures in the English National Health Service: A documentary analysis [file sj-pdf-4-hsr-10.1177_13558196221091518.pdf]

SUPPLEMENT 4

S4: Details pertaining to each policy's date, review date, and changes/iterations to policy criteria observed over the course of the study

Clinical Procedure	Number of policies analysed	Policy version number (if known) and date (month/year)	Date of policy reviews and iterations (where stated)	Had the policy criteria themselves changed over the course of the study? (Checked 28 March 2020)	Potentially relevant NICE guidance consulted
**Subacromial decompression**	Region-1	V1617.1.03, November 2016	Intended: November 2016<br>Actual: rebranding March 2018, References for smoking/BMI/PALS and branding updated in October 2018; criteria reviewed/amended in April 2019.	Changed in April 2019	British Elbow & Shoulder Society (BESS), British Orthopaedic Association (BOA), Royal College of Surgeons for England (RCSEng) Commissioning guide: Subacromial Shoulder Pain (November 2014)
	Region-11	June 2017	Intended: June 2020.<br>Policy unchanged.	Unchanged	
	Region-12	V0.2, January 2018	N/A V0.3 May 2018; V0.4 July 2018; V0.5 Feb 2019; V1.0 March 2019; V1.1 Aug 2019	Changed in February 2019	
	Region-2	V1, November 2015	Intended: November 2017, but new policy not available until July 2018. The policy wording unchanged.	Unchanged	
**Hip Replacement**	Region-1	1617.1.01, June 2016	Intended: June 2019;<br>Actual: rebranding March 2018, References for smoking/BMI/PALS and branding updated in October 2018; criteria reviewed/amended in April 2019.	Changed in April 2019	NICE CG177 Osteoarthritis: care and management (February 2014)<br><br>NICE IPG363: Minimally invasive total hip replacement (October 2010)

	Region-8	August 2017	N/A<br>Changes to join policy in April 2019, but no changes to hip replacement	Unchanged	NICE TA304: Total hip replacement and resurfacing arthroplasty for end-stage arthritis of the hip (February 2014)
	Region-9	1.0, December 2017	Intended: June 2020	Unchanged	
	Region-10	April 2016	N/A<br>Updated in April 2019, but criteria for hip replacement unchanged.	Unchanged	
	Region-3	April 2016	N/A<br>Updated January 2020, but criteria unchanged	Unchanged	
**Knee arthroscopy**	Region-1	1617.1.01, June 2016	Intended: June 2019;<br>Actual: rebranding March 2018, References for smoking/BMI/PALS and branding updated in October 2018; criteria reviewed/amended in April 2019.	Changed in April 2019	NICE CG177: Osteoarthritis: care and management (February 2014)<br><br>NICE IPG493: Arthroscopic radiofrequency chondroplasty for discrete chondral defects of the knee (May 2014)<br><br>NICE IPG162: Mosaicplasty for knee cartilage defects (March 2006)<br><br>NICE IPG430: Partial replacement of the meniscus of the knee using a biodegradable scaffold (July 2012)<br><br>NICE IPG474: Arthroscopic trochleoplasty for patellar instability (January 2014)<br><br>NICE IPG560: Microstructural scaffold (patch) insertion without autologous cell
	Region-3	V4, April 2016	N/A<br>Updated January 2020, but criteria unchanged	Unchanged	
	Region-4a	April 2017	N/A<br>Name of CCG changed to Region-4a in July 2018- policy criteria unchanged.<br>V3.1 in May 2019, to reflect EBI criteria. Criteria changed.	Changed in May 2019	
	Region-5	April 2017	Joint policy for mutliple conditions, but knee arthroscopy criteria not reviewed until April 2019. Unchanged.	Unchanged	

	Region-2	November 2015	Due to be reviewed November 2017; new policy came out in July 2018 (identified on 28 March 2020), but not available when checked online in August 2018.	Unchanged	implantation for repairing symptomatic chondral knee defects (June 2016)<br><br>NICE IPG230– Arthroscopic knee washout, with or without debridement, for the treatment of osteoarthritis (August 2007)<br><br>NICE TA477: Autologous chondrocyte implantation for treating symptomatic articular cartilage defects of the knee (October 2017)<br><br>NICE TA508: Autologous chondrocyte implantation using chondrosphere for treating symptomatic articular cartilage defects of the knee (March 2018)<br><br>NICE MIB30: LARS for reconstructing damaged intra-articular cruciate knee ligaments (May 2015)
**Hip arthroscopy**	Region-1	1617.1.01, June 2016	Intended: June 2019;<br>Actual: rebranding March 2018, References for smoking/BMI/PALS and branding updated in October 2018; criteria reviewed/amended in April 2019.	Changed in April 2019	NICE IPG408: Arthroscopic femoro–acetabular surgery for hip impingement syndrome (September 2011)
	Region-6	March 2012	Intended: March 2013, but appears to be unchanged. When checked on 28 March 2020, names of CCGs changed by policy no different.	Unchanged	
	Region-4a CCG	May 2017	Intended: April 2018, but not changed or reviewed. Name of CCG changed to Region-4a in July 2018, but no changes at all to hip arthroscopy criteria.	Unchanged	

	Region-4b	December 2016	Intended: December 2019<br>Joint policy on multiple procedures updated in March 2019, but criteria/policy for hip arthroscopy completely unchanged.	Unchanged	
**Rotator cuff repair**	Region-1	1617.1.03, November 2016	Intended: November 2016<br>Actual: rebranding March 2018, References for smoking/BMI/PALS and branding updated in October 2018; criteria reviewed/amended in April 2019.	Changed in April 2019	NICE Clinical Knowledge Summary: Shoulder pain (Scenario: Rotator cuff disorders) (April 2017)<br><br>British Elbow & Shoulder Society (BESS), British Orthopaedic Association (BOA), Royal College of Surgeons for England (RCSEng) Commissioning guide: Subacromial Shoulder Pain (2014)
	Region-2	V1, November 2015	Intended: November 2017, but new policy not available until July 2018. The policy wording unchanged.	Unchanged	
	Region-14	January 2018	Intended: January 2021<br>Reviewed in March 2019, where subacromial decompression references removed. Criteria for rotator cuff repair completely unchanged.	Unchanged	
**Surgery for Dupuytren's contracture**	Region-1	1718.3.01, December 2017	Intended: November 2020<br>Actual: rebranding March 2018, References for smoking/BMI/PALS and branding updated in October 2018; criteria reviewed/amended in April 2019.	Changed in April 2019	NICE Clinical Knowledge Summary: Dupuytren's disease (November 2015)<br><br>NICE TA459: Collagenase clostridium histolyticum for treating Dupuytren's contracture (July 2017)<br><br>NICE IPG43: Needle fasciotomy for Dupuytren's contracture (February 2004)
	Region-3	V4, April 2016	Updated (V5) in April 2019 but wording unchanged; updated again in January 2020 and wording changed.	Changed in January 2020	
	Region-4a CCG	May 2017	Intended: April 2018, but not changed or reviewed. Name of CCG changed to Region-4a in July 2018. Criteria for Dupytren's contracture completely unchanged.<br>Changed in May 2019 - policy criteria changed	Changed in May 2019	

	Region-4b	December 2016	Intended: December 2019<br>Joint policy on multiple procedures updated in March 2019, with changes to dupuytren's contracture policy.	Changed in March 2019	
	Region-2	February 2014	Due to be reviewed February 2016; new policy came out in March 2018 (identified on 28 March 2020), but not available when checked online in August 2018.	Changed in March 2018, but this latest policy was not identified when searched in August 2018.	
	Region-6	September 2017	Updated in October 2019, and then again in March 2020.	Changed in October 2019	
**Surgery for Trigger finger**	Region-1	1617.1.01, February 2017	Intended: February 2020<br>Actual: rebranding March 2018, References for smoking/BMI/PALS and branding updated in October 2018; criteria reviewed/amended in April 2019.	Changed in April 2019	No NICE guidance identified
	Region-3	V4, September 2016	Updated (V5) in April 2019 but wording unchanged; updated again in January 2020 and wording changed.	Changed in January 2020	
	Region-4b	December 2016	Intended: December 2019<br>Joint policy on multiple procedures updated in March 2019, with changes to trigger finger policy.	Changed in March 2019	
	Region-13	October 2014	Updated October 2019.	Changed in October 2019	
	Region-2	November 2017	Intended: November 2019, but policy unchanged from original	Unchanged	

**Knee Replacement**	Region-1	November 2016	Intended: November 2019<br>Rebranding March 2018; References for smoking/BMI/PALS and branding updated in October 2018; criteria reviewed in May 2019 but unchanged. Policy wording unchanged from original.	Unchanged	NICE CG177: Osteoarthritis: care and management (February 2014)<br><br>NICE IPG345: Mini-incision surgery for total knee replacement (May 2010)
	Region-3	V4, September 2016	Two subsequent versions with cosmetic differences in April 2019 and December 2020, but policy content identical to original.	Unchanged	
	**Region-7**	V6, April 2018	V7 April 2019 and V8 April 2020 - criteria for knee replacement unchanged from original policy.	Unchanged	
