# Supplementary material for: Variations in policies for accessing elective musculoskeletal procedures in the English National Health Service: A documentary analysis
Source: J Health Serv Res Policy. 2022 May 15;27(3):190–202. doi: 10.1177/13558196221091518 (PMC9277328; doi:10.1177/13558196221091518)
Supplement: Supplemental Material - Variations in policies for accessing elective musculoskeletal procedures in the English National Health Service: A documentary analysis [file sj-pdf-6-hsr-10.1177_13558196221091518.pdf]

Journal of Health Services Research & Policy
Variations in policies for accessing elective musculoskeletal procedures in the English National Health Service: A documentary analysis
Leila Rooshenas L, Ijaz S, Richards A, Realpe A, Savovic J, Jones T, Hollingworth W and Donovan J

SUPPLEMENT 6

S6: Policies for Dupuytren's contracture: extracts relating to threshold modifiers		
**Region-4a (highest spend)**	**Region-4b (highest spend)**	**Region-6 (second from highest spend)**
Surgery of Dupuytren's contracture will only be funded in accordance with the criteria specified below:<br>- Flexion deformity >30° at the MCPJoint or PIPJoint<br>**OR**<br>- Rapidly progressive disease<br>**OR**<br>- Contracture interferes with lifestyle and/or occupation	For criteria for referral for surgical intervention follow the guidance issued within the NICE produced Clinical Knowledge Summary for Dupuytren's disease.	Surgical treatment for Dupuytren's contracture will be routinely commissioned in [Region] in the following circumstances:<br>- Metacarpophalangeal joint contracture of 30 degrees or more (inability to put hand flat on table)<br>**OR**<br>- Any degree of proximal interphalangeal joint contracture<br>**OR**<br>- Significant functional impairment<br>**OR**<br>- Where there is significant threat to hand function due to Dupuytren's diathesis
**Region-1 (higher than average spend)**	**Region-2 (lower than average spend)**	**Region-3 (lowest spend)**
The CCG will agree to fund surgical intervention for Dupuytren's where the following criteria<br>have been met:<br>**1.** The patient has a 30 degree, or greater, fixed flexion deformity at either the metacarpophalanageal joint or proximal interphalangeal joint.<br>**AND**<br>**2. a)** The patient cannot flatten their fingers or palm on a table.<br>**OR**<br>**b)** There has been rapid progression over a few months.<br>**OR**<br>**c)** The patient is suffering from significant functional impairment*<br>**Significant functional impairment is defined by [Region] as:*<br>*- Symptoms preventing the patient fulfilling routine work or educational responsibilities*<br>*- Symptoms preventing the patient carrying out routine domestic or carer activities*	The CCG will ONLY fund the surgical treatment of Dupuytren's Contracture according to the following criteria:<br>**-**The patient has a **moderate** to **severe** form of disease as defined below.<br><br><u>Classification for Severity of Dupuytren's Contracture:</u><br>Mild: No functional impairment. Contractures less than 30° at metacarpophalangeal joints (MCPJ)<br>**Moderate**: Notable functional impairment 30-60° fixed flexion at the MCPJ and less than 30° at the proximal interphalangeal joint (PIPJ)<br>**Severe**: Fixed flexion greater than 60° at the MCPJ and greater than 30° at the PIPJ	[Named] CCGs will fund surgery for patients with either:<br>**1.** A fixed flexion deformity >30° at the MCP Joint or PIP Joint.<br>**OR**<br>**2.** A fixed flexion deformity between 0 and 30 degrees at the PIPJ where either there is<br>**-** Rapidly progressive disease<br>**OR**<br>**-** A contracture that interferes with lifestyle and/or occupation
